# Multidisciplinary Management of Obstructed Hemivagina and Ipsilateral Renal Anomaly (OHVIRA) Syndrome in an Adolescent With Complex Congenital Anomalies: A Case Report

**DOI:** 10.7759/cureus.56961

**Published:** 2024-03-26

**Authors:** Munara K Kerimkulova, Sergio Rodrigo Oliveira Souza Lima, Ayah E Elamin, Yusra H Hamid, Nuzhat Faran, Ismail Khan

**Affiliations:** 1 Internal Medicine, Kyrgyz State Medical Academy, Bishkek, KGZ; 2 Plastic Surgery, Hospital da Bahia, Salvador, BRA; 3 Anatomy, National University-Sudan, Khartoum, SDN; 4 Community Medicine, University of Khartoum, Faculty of Medicine, Khartoum, SDN; 5 Internal Medicine, Fatima Memorial Hospital, Lahore, PAK; 6 Internal Medicine, Nishtar Medical University, Multan, PAK

**Keywords:** gyn pathology, surgical management, multidisciplinary approach, congenital anomalies, ohvira syndrome

## Abstract

Obstructed hemivagina and ipsilateral renal anomaly (OHVIRA) syndrome presents with complex diagnostic and therapeutic challenges and is characterized by uterine didelphys, obstructed hemivagina, and ipsilateral renal anomaly. A 14-year-old female with a history of anorectal malformation and urogenital sinus anomaly presented with menstrual blood in her urine, abdominal pain, and distension. Investigations revealed a bicornuate uterus, vesicovaginal fistula, and right ovarian cyst, leading to the diagnosis of OHVIRA syndrome. A multidisciplinary approach resulted in salpingo-oophorectomy and subtotal hysterectomy.

This case highlights the diagnostic challenges and emphasizes the role of advanced imaging and a multidisciplinary team in managing such complex conditions. It stresses the importance of patient-centered surgical planning tailored to the individual's anatomy and reproductive goals. Early recognition and a tailored, multidisciplinary approach are crucial in managing OHVIRA syndrome and improving outcomes for patients with rare congenital anomalies.

## Introduction

In the evolving landscape of pediatric and adolescent gynecology, managing congenital reproductive tract anomalies presents a significant clinical challenge, demanding a nuanced understanding of their complex presentations and implications for affected individuals. Among these anomalies, obstructed hemivagina and ipsilateral renal anomaly (OHVIRA) syndrome stands out due to its rarity and the intricate diagnostic and therapeutic approaches it necessitates. OHVIRA syndrome, also known as Herlyn-Werner-Wunderlich syndrome, embodies a spectrum of Müllerian duct anomalies, typically manifesting with symptoms such as pelvic pain, menstrual irregularities, and sometimes acute urinary retention, primarily during menarche [1,2]. The condition underscores the critical interplay between embryological development and clinical presentation, highlighting the importance of early diagnosis and intervention to prevent complications such as endometriosis, renal dysfunction, and issues related to fertility [3].

The multidisciplinary management of OHVIRA syndrome, involving gynecologists, radiologists, urologists, and sometimes pediatric surgeons, emphasizes the necessity of a collaborative approach to care. This collaboration is pivotal in navigating the diagnostic complexities and determining the most appropriate therapeutic strategies, ranging from conservative management to surgical intervention, depending on the individual's symptoms, anatomy, and reproductive desires [4]. The role of advanced imaging techniques, particularly magnetic resonance imaging (MRI), cannot be overstated, offering detailed anatomical insights that guide surgical planning and patient counseling [2].

This case report aims to delineate the diagnostic journey and therapeutic management of a patient with OHVIRA syndrome, underscoring the diagnostic challenges and the critical role of a patient-centered, multidisciplinary approach in achieving optimal outcomes. Through this case, we aim to contribute to the existing literature on OHVIRA syndrome, offering insights into effective diagnosis and management strategies and highlighting the importance of early intervention to mitigate long-term complications and enhance the quality of life for patients with this complex condition.

## Case presentation

A 14-year-old female with a history of anorectal malformation (ARM) and urogenital sinus anomaly underwent early interventions, including a sigmoid loop colostomy at one month and anterior sagittal anorectoplasty at one year, to manage her conditions. Years later, she exhibited symptoms of abdominal distension and pain, attributed to hydrometrocolpos, and managed by drainage.

The current presentation is marked by a new and concerning symptom of menstrual blood in urine, noted for approximately three days of each month, coinciding with her menstrual period. This symptom is accompanied by recurrent episodes of abdominal pain, prompting her to visit the gynecology department for further evaluation and management.

Upon physical examination, the patient appeared to be in no acute distress. Notably, a scar from a previous stoma closure was visible on the abdomen, indicative of her surgical history. Upon inspection, the abdomen was soft and non-rigid but revealed distension in the pelvic region. Palpation of the abdomen elicited tenderness, particularly on deep palpation, suggesting an underlying pelvic pathology without signs of peritonitis.

The genital examination revealed well-developed labias. Two distinct openings were appreciated at the vestibule: presumably the urethral meatus, and the other likely representing an abnormal communication or remnant of the urogenital sinus anomaly, further complicating her congenital condition. No erythema, discharge, or external mass was noted, and the external genitalia was otherwise normal in appearance. The remainder of the physical examination, including cardiovascular, respiratory, and neurological systems, was unremarkable, with no additional abnormalities detected that would contribute to her current gynecological presentation.

Laboratory tests indicated a slight elevation in activated partial thromboplastin time (aPTT) at 33 seconds and alanine transaminase (ALT) at 53 U/L, mild anemia with a hemoglobin of 12.3 g/dL, and normal renal function and electrolytes, with urea at 22.98 mg/dl and creatinine at 0.73 mg/dl. These findings suggest minor deviations without significant clinical concerns, guiding further evaluation of her complex presentation.

**Figure 1:**
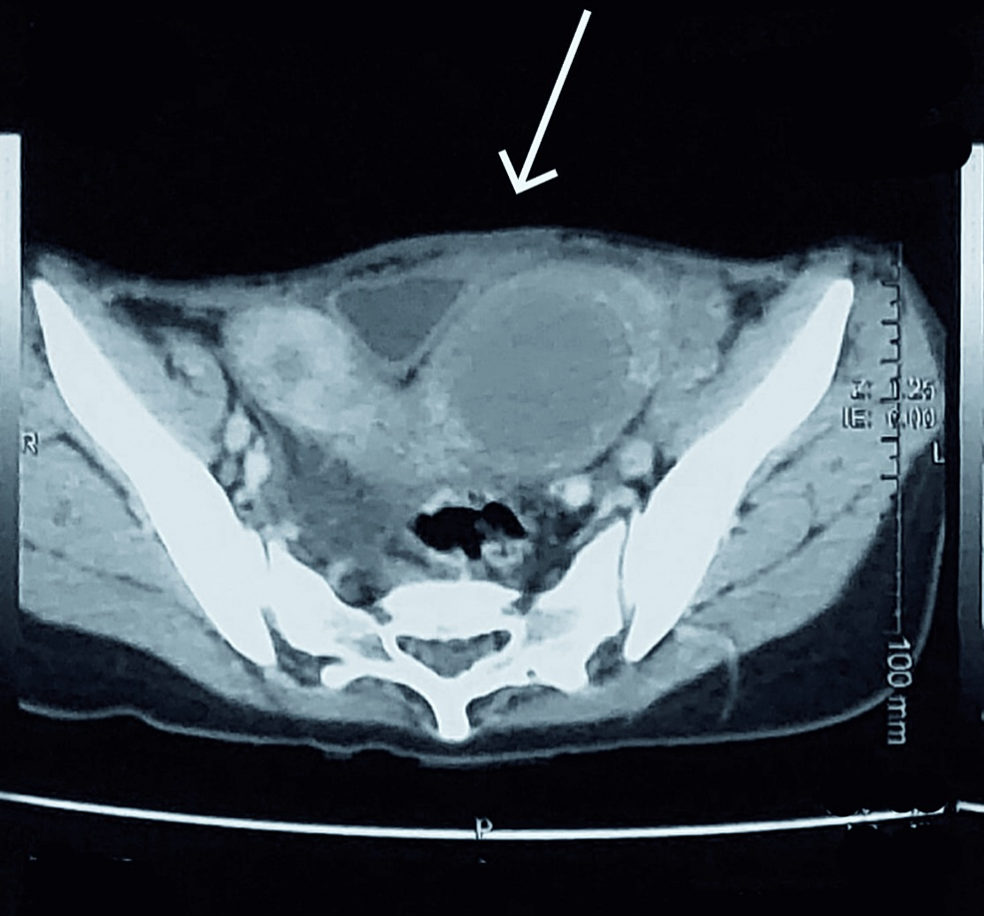
CT scan illustrating bicornuate uterus, pointed with an arrow. CT scan: computed tomography scan

Imaging studies provided critical insights into the patient's complex condition. An ultrasound examination displayed a bicornuate uterus with debris fluid suggestive of hydrometrocolpos and a multiseptated large cyst on the right ovary. The micturating cystourethrogram (MCUG) revealed a vesicovaginal fistula, explaining the presence of menstrual blood in the urine. Computed tomography (CT) of the abdomen and pelvis confirmed the presence of a bicornuate uterus with fluid accumulation in its cavities and a left adnexal cyst alongside a hypoplastic but normal-looking right kidney. Notably, the CT scan also identified a limbous vertebra at L5 and atelectatic bands in the right lower lobe of the chest, highlighting additional vertebral and pulmonary anomalies. A CT scan of the pelvis is provided in Figure 1.

**Figure 2:**
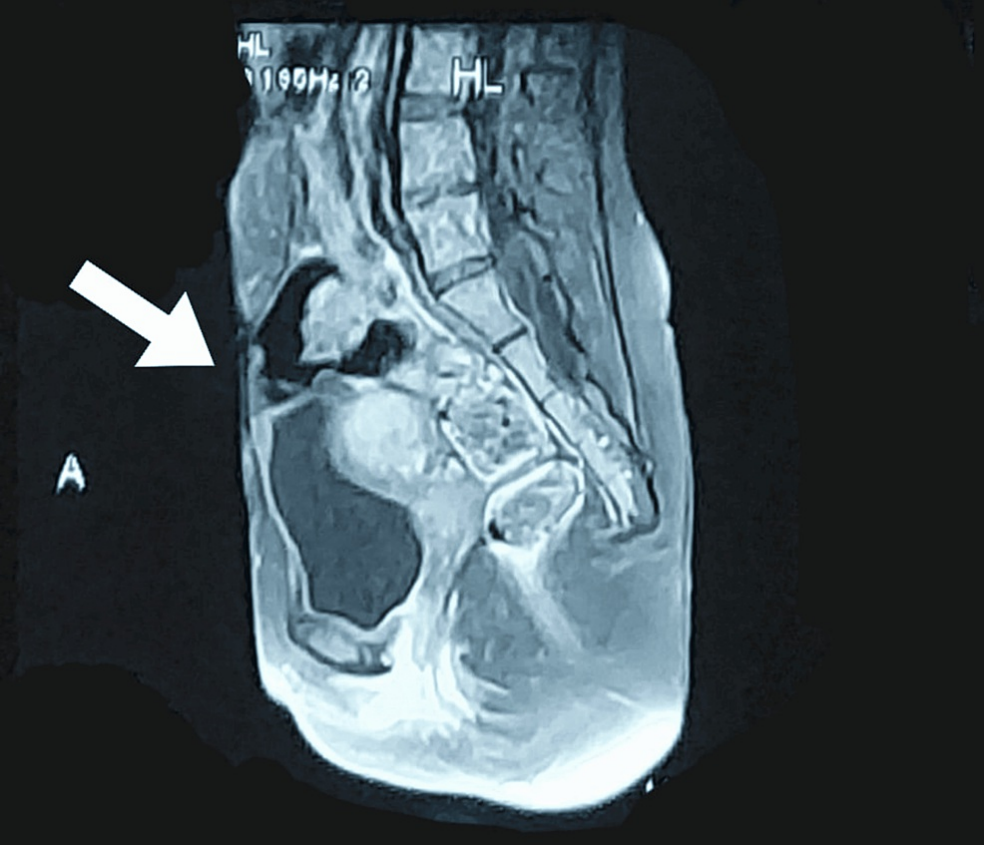
MRI pelvis showing bicornuate uterus, as pointed by the arrow. MRI: magnetic resonance imaging

Magnetic resonance imaging (MRI) of the pelvis further detailed the uterine anomaly, showing a normal-looking right horn and a dilated left horn with partial to complete cervical canal stenosis alongside a right para-ovarian cyst measuring 31*15.4mm. These findings were instrumental in diagnosing the patient's condition and guiding the subsequent management and intervention strategies. The MRI image is provided in Figure 2.

The differential diagnosis for a patient presenting with menstrual blood in urine, abdominal pain, and a history of congenital anomalies includes a spectrum of conditions affecting the reproductive tract's structure and function. Imperforate hymen, transverse vaginal septum, and cervical atresia were considered due to their potential to obstruct menstrual flow, leading to symptoms of pain and distension. These conditions could theoretically cause retrograde menstruation or unusual presentations of menstrual blood but typically do not explain urinary involvement. Endometriosis was another consideration, given its association with pelvic pain and the potential for ectopic endometrial tissue to cause unusual bleeding patterns; however, it seldom results in menstrual blood in urine without significant involvement of the urinary tract. Congenital uterine anomalies were also on the list of differentials due to the patient's known history of anorectal malformation and urogenital sinus, suggesting a broader spectrum of developmental abnormalities.

The diagnosis was refined after thorough evaluation, including detailed imaging studies revealing a bicornuate uterus with hydrometrocolpos, a vesicovaginal fistula on MCUG, and a multiseptated large cyst on the right ovary. The constellation, particularly the bicornuate uterus, in conjunction with a urogenital anomaly and the presence of a vesicovaginal fistula, led to the diagnosis of OHVIRA syndrome (obstructed hemivagina and ipsilateral renal anomaly), along with a right ovarian cyst. OHVIRA syndrome, a rare congenital condition characterized by a triad of uterovaginal duplication, obstructed hemivagina, and ipsilateral renal anomalies, perfectly encapsulates the patient's clinical presentation and imaging findings. The identification of a right ovarian cyst adds complexity to her condition, necessitating specific attention in her treatment plan.

A multidisciplinary team approach was adopted following a comprehensive diagnostic evaluation to address the patient's complex condition. The patient and her parents were thoroughly counseled regarding the nature of her condition, potential treatment options, and anticipated outcomes, ensuring informed consent was obtained for the proposed surgical intervention.

The patient underwent an examination under anesthesia (EUA) to facilitate a detailed intraoperative assessment of her reproductive and urinary tract anomalies. A diagnostic laparoscopy was initially performed, allowing for a minimally invasive exploration to confirm the extent of the congenital anomalies and plan the surgical approach. Subsequently, a laparotomy was undertaken to provide direct access to the pelvic organs.

A right salpingo-oophorectomy addressed the multiseptated large cyst on the right ovary, aiming to prevent potential complications such as torsion, rupture, or malignancy. A urologist was integral to the surgical team, performing Double J (DJ) stenting of the ureter before surgery to safeguard ureteral integrity and ensure postoperative urinary tract function.

The laparotomy revealed two separate horns of the uterus sharing a common cervix, consistent with the patient's diagnosis of OHVIRA syndrome. The left uterine horn, which had no connection to the vagina and contributed to the patient's symptoms of hydrometrocolpos and menstrual blood in urine, was surgically removed. Efforts were made to create an outflow tract for the right uterine horn by dissecting a transverse vaginal septum. However, this endeavor faced prognostic challenges, as discussed with a consulting plastic surgeon, due to the complex nature of the patient's congenital anomalies and the risk of compromising adjacent structures.

Given the adherence of the urinary bladder to the cervix and the associated risk of recurrent complications, a decision was made for a subtotal hysterectomy. This procedure aimed to alleviate the patient's symptoms and prevent future complications related to the obstructed and malformed uterine structure. Post-operatively, the urologist meticulously evaluated the bladder's integrity to ensure no iatrogenic injury had occurred during the intervention.

After completing the surgical procedures, the patient was transferred to the postoperative care unit, where she received continuous monitoring to assess her recovery and immediate response to the surgery. Initial postoperative care focused on pain management, infection prevention, and monitoring for signs of urinary retention or dysfunction due to the proximity of the surgical sites to the urinary tract. The patient was administered intravenous antibiotics, specifically ceftriaxone 1g twice daily for five days, to mitigate the risk of postoperative infections. Additionally, paracetamol (1 g) was given intravenously twice a day for five days to manage pain and inflammation. Attention was also given to ensuring the patient's emotional and psychological well-being, considering the extensive nature of the surgery and its implications for her future health and fertility.

The patient demonstrated satisfactory progress in her recovery, with no immediate postoperative complications. The Double J (DJ) stent's status and the functionality of the remaining ovarian and urinary structures were periodically assessed through follow-up imaging studies. Upon meeting the criteria for discharge, including stable vital signs, pain control, and the ability to perform basic activities of daily living, the patient was discharged with a comprehensive home care plan. This included oral antibiotics, ciprofloxacin 500mg twice daily for 14 days, and paracetamol, with instructions to take two tablets for pain management. Instructions were provided for wound care, signs of infection, and when to seek immediate medical attention.

A follow-up appointment was scheduled two weeks post-discharge to assess wound healing, evaluate the patient's overall recovery, and discuss the long-term management plan. Future follow-ups would focus on monitoring renal function, the health of the remaining reproductive organs, and addressing any psychological or emotional support needs. The multidisciplinary team remained involved in her care, ensuring a holistic approach to her rehabilitation and adjustment post-surgery, with particular attention to her reproductive health and potential fertility considerations moving forward.

## Discussion

This case report delineates the challenges and strategic interventions in managing OHVIRA syndrome in a 14-year-old female, further complicated by the presence of an ovarian cyst. OHVIRA syndrome, characterized by obstructed hemivagina and ipsilateral renal anomaly, is a rare yet impactful congenital condition that demands a nuanced diagnostic and therapeutic approach due to its complex interplay with other gynecological conditions. Incorporating a multidisciplinary team and leveraging advanced imaging techniques such as MRI is crucial for a tailored and effective management strategy, emphasizing the syndrome's diagnostic challenges and the need for personalized care [5-7].

OHVIRA syndrome's clinical manifestation is diverse, ranging from cyclic pelvic pain to dysmenorrhea, often exacerbated by menstrual blood accumulation in an obstructed hemivagina. The case's complexity is heightened by the patient's concurrent anorectal malformation (ARM) and urogenital sinus anomaly, illustrating the intricate diagnostic landscape where symptoms such as menstrual blood in urine can mislead clinicians [5]. Surgical intervention, including salpingo-oophorectomy and subtotal hysterectomy, was necessitated not only by the ovarian cyst but also to alleviate the syndrome's symptomatic burden and mitigate potential complications, aligning with literature that advocates for a surgical resolution to enhance life quality [3,5].

The pivotal role of individualized surgical planning, underscored by the patient's anatomy, symptomatology, and reproductive aspirations, highlights the indispensable value of a multidisciplinary approach. This strategy ensures comprehensive care that spans gynecology, urology, and pediatric surgery, aiming for optimal patient outcomes [5,6]. Alternative management strategies, focusing on conservative management and reproductive tract preservation, reflect the overarching principle of patient-centered care tailored to individual patient profiles and long-term prognoses [5,6].

Early and accurate diagnosis emerges as a cornerstone in managing OHVIRA syndrome, with advanced imaging modalities playing a critical role in unveiling the patient's complex anatomy and facilitating a strategic surgical approach. This proactive diagnostic effort is pivotal in averting complications, preserving renal function and fertility, and circumventing the psychological distress associated with delayed treatment [6].

This case exemplifies the diagnostic and management intricacies of OHVIRA syndrome, compounded by overlapping symptoms with other gynecological conditions. A thorough clinical evaluation, augmented by advanced imaging techniques, is indispensable for a timely and accurate diagnosis, underscoring the need for heightened clinical awareness among healthcare providers [2,5].

The comprehensive management of this case through a multidisciplinary lens underscores the importance of collaborative expertise across gynecology, urology, and radiology. This collaborative effort facilitated a nuanced evaluation and treatment plan. It highlighted the essence of multidisciplinary care in enhancing decision-making, surgical planning, and postoperative care, ultimately culminating in improved patient outcomes and satisfaction [4].

The management of OHVIRA syndrome, particularly in the context of concurrent congenital anomalies, accentuates the critical need for a multidisciplinary, patient-centered approach. Early diagnosis, informed by advanced imaging and collaborative expertise, enables tailored surgical interventions that address symptomatic relief and long-term health outcomes. This case reinforces the imperative of integrating clinical vigilance with a holistic care model to optimize the quality of life for patients navigating complex gynecological conditions [8,9].

While our case report provides comprehensive insights into the management of OHVIRA syndrome in an adolescent with complex congenital anomalies, it is important to acknowledge certain limitations. Specifically, intraoperative and postoperative images were unavailable due to inherent complexities in the surgical intervention and logistical constraints encountered during postoperative care. Despite these limitations, the detailed description of the diagnostic journey, therapeutic management, and multidisciplinary approach serves to enhance understanding and guide future research and clinical practice in managing similar cases.

## Conclusions

The intricate management of this case illuminates the essential need for an inclusive diagnostic lens when addressing adolescent pelvic pain and menstrual irregularities, with a particular focus on rare congenital anomalies like OHVIRA syndrome. It exemplifies the paramount importance of a patient-centered surgical approach, underpinned by a multidisciplinary team's expertise, to tailor treatment strategies that respect individual patient desires and health outcomes. This case serves as a compelling reminder of the critical role of advanced imaging in early diagnosis and the necessity of engaging patients and their families in the treatment journey, ensuring informed consent, and aligning care with patient expectations. The lessons from this case advocate for a holistic, informed, and empathetic approach in clinical practice to optimize outcomes and enhance the quality of life for patients with complex gynecological conditions.
